# Health care contact following a new incident neck or low back pain episode in the general population; the HUNT study

**DOI:** 10.1186/s12913-016-1326-5

**Published:** 2016-03-08

**Authors:** Astrid Woodhouse, Kristine Pape, Pål R. Romundstad, Ottar Vasseljen

**Affiliations:** Department of Public Health and General Practice, Faculty of Medicine, Norwegian University of Science and Technology, Medisinsk teknisk forskningssenter, P.O. Box 8905, Trondheim, 7491 Norway; National Competence Centre for Complex Symptom Disorders, St Olav’s Hospital, Trondheim, Norway

**Keywords:** Neck pain, Low back pain, Health care seeking, Health care utilization, Health care contact

## Abstract

**Background:**

Low back and neck pain are commonly reported in the general population and represent frequent causes for health care consultations. The main aim of this study was to describe the determinants of health care contact during a 1-year period in a general population with recent onset spinal pain.

**Methods:**

From 9056 participants in a general health survey in Norway we identified 219 persons reporting a recent onset (<1 month) of low back or neck pain. Questionnaires were given at 1 (baseline), 2, 3, 6 and 12 months after pain debut. The main outcome was self-reported health care contact due to spinal pain. Associations between health care contact and pain-related factors, other somatic and mental health factors, pain-related work limitations, physical activity and sociodemographic factors were explored.

**Results:**

Conventional health care was sought by 93 persons (43 %) at least once throughout the year following the onset of pain. 18 persons (8 %) sought alternative health care only and 108 persons (49 %) sought no kind of health care. Baseline reports of coexisting low back and neck pain of equal intensity, poor self-reported health, symptoms of anxiety or depression, obesity and smoking were all associated with an increased tendency to seek conventional health care. Pain intensity and pain-related work limitations at each occasion were strongly associated with concurrent health care contact throughout the year. Higher education was associated with a reduced tendency to contact health care and no association was found for physical activity.

**Conclusion:**

The main finding in this study was that people from the general population who seek health-care for a new incident of neck or low back pain report more symptoms of mental distress, poorer self-reported health and more intense pain with stronger work limitations compared to those who do not. The findings suggest that identification of complementary symptoms is highly relevant in the examination of spinal pain patients, even for those with recent onset of symptoms.

## Background

Low back pain (LBP) and neck pain (NP) are frequently reported in the general populations [1, 2]. In spite of the self-limiting nature of most spinal pain conditions and small effects of commonly applied treatment interventions [3–6], many spinal pain sufferers will consult professional help for their problems with frequent visits to primary care clinics [7, 8].

In a recent article exploring the natural course of acute neck and low back pain in the general population we found a rapid decrease of pain within the first month [6]. The natural course of pain was, in fact, remarkably similar to the course of pain described in clinical studies with patients receiving treatment [3]. We were also surprised to find that the proportion who had sought help for their new pain episode was remarkably stable around 20 % at each of the five assessment occasions throughout the following year [6]. The proportion seeking help was much higher among those who reported equally intense pain in the neck and the low back regions compared to those with pain in either site. The question arose whether care-seeking was linked to the pain episode itself or to other health or personality issues.

To understand why some people seek health care throughout the course of a pain situation is important, whether the pain is acute, chronic or intermittent. From the clinicians’ perspective, such knowledge may be helpful in the planning and performing of optimal treatment as well as gaining knowledge about preventive strategies. Our understanding of factors and characteristics of people seeking health care is still limited, particularly the knowledge on how those that choose to seek health care differ from those who do not. Some previous studies have described clinical populations, and thus only include the care-seekers [9]. A few general population surveys have investigated decisive factors for seeking health care including symptom related factors, socio-demographic factors, lifestyle factors and general health [10–12], but their findings are inconsistent except for repeatedly positive associations between care-seeking and higher levels of pain and work limitations. Psychological distress has been associated with care-seeking in persons with chronic widespread pain; a condition where spinal pain by definition is required [13]. However, the cross-sectional designs and the variation in study populations still leave us with limited understanding of care-seeking behaviour in spinal pain conditions.

In this population based study we hold data on sociodemography, somatic and mental health, dimensions of pain, pain-related work limitations and health care contact at baseline and throughout a year following a new spinal pain episode. The main aim of this study was thus to prospectively study the determinants of health care contact during 1-year follow-up in a population with recent onset spinal pain.

## Methods

### Study design and population

Data for this prospective cohort study were obtained from two of 24 municipalities that participated in the third wave of the Norwegian Nord-Trøndelag Health Study 2006–08 (HUNT 3) [14]. All inhabitants of Nord-Trøndelag County aged 20 years or older were invited to HUNT 3, and 50 807 (54.1 %) participated.

A total of 9 056 subjects aged 20–67 years were screened for eligibility for the current study. Three questions were used to identify the study cohort: ^1)^ “Do you have pain in your shoulder-neck area or low back today?” ^2)^ “Is it less than one month since the pain started?” and ^3)^ “Were you without this pain the 3 months previous to last month?”. Subjects answering “yes” to all three questions were invited to participate in the current study. Signed informed consent was obtained and the study was approved by the Regional Committee for Medical Health Research Ethics of Central Norway (REC Central). The study population has been thoroughly described elsewhere [6].

### Data collection

**Fig. 1:**
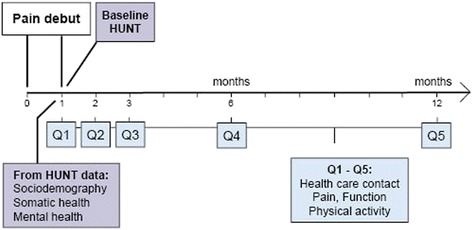
The study timeline

Data were collected by questionnaires at 1 (baseline), 2, 3, 6 and 12 months after pain debut (Fig. 1). The baseline measurement at 1 month represents the point in time when the subjects attended HUNT 3 and where the new pain episode had appeared within the last month. The comprehensive questionnaires for the entire HUNT 3 population as well as a brief 18-item questionnaire designed for the study cohort were presented to the subjects at the health survey location site (Q1, Fig. 1). In the main HUNT 3 survey, questionnaires where partly answered before presentation at the screening site and partly on-site. This resulted in lower response rate on-site and thereby more missing values on the baseline health variables taken from this part (anxiety, depression and insomnia). The brief questionnaire was also used in the follow-up period and sent by post at 2, 3, 6 and 12 months (Q2 – Q5, Fig. 1). It included questions on various dimensions of pain, use of pain medication (last week), sick leave status (at present), pain-related work limitations, physical activity and the type of health care consultations the subjects attended during the previous month.

### Outcome variables (health care contact)

The subjects were asked at all five occasions (Q1-Q5) whether they had received treatment for their spinal pain from any kind of health care provider within the last month. If they replied positively, they were also asked to report what kind of health care provider(s) they had seen. The seven options for health care providers were physician, psychologist, physiotherapist, chiropractor, osteopath/naprapath, homeopath/acupuncturer/other alternative treatment or other health care provider(s). Treatment by health care providers was categorized into two subcategories; conventional health care (physician, physiotherapist, chiropractor and psychologist) and alternative health care (including osteopath/naprapath, homeopath/acupuncturer/other alternative treatment and other health care provider). Those who reported both conventional and alternative treatment were included in the conventional health care group. Those who reported using prescribed medication or being on sick leave were also included in the “physician” group, as direct report of contact with the physician was under-reported (prescribed medication or sick leave compensation requires contact with a physician in Norway). The main outcome variable in the analyses was whether or not the subjects consulted conventional health care. Those who sought both conventional and alternative care were included in this group. Similar analyses on the subjects consulting alternative health care only were not possible due to the small sample size. The exact number of consultations from each health care provider was not available, only whether or not there had been contact during the previous month.

### Predictor variables recorded at baseline

#### Pain related factors

Current pain intensity and the strongest pain intensity last month were each reported on a 0–10 Numeric Rating Scale (NRS) with checkbox 0 labeled “no pain” and 10 labeled “worst imaginable pain”. Pain-related work limitations were measured by one question regarding the intensity of pain when doing work related tasks (five point scale) and the frequency of the pain (three point scale) which added up to a 0–6 point scale. High score indicated higher pain burden and stronger work limitations due to pain [15]. Two variables for pain location were registered: the spinal pain location in three categories (low back pain, neck pain, and equally intense pain in both low back and neck) and the total number of musculoskeletal pain sites (max 12 sites) [16]. The frequency of previous pain episodes was registered (no previous episodes, once a year or less, two or more per year). Finally, the reported use of pain medication during the previous week was registered, in which the subjects could also checkmark different over-the-counter and prescription pain medications.

#### Other health related factors

Health related factors in this study included somatic health and mental distress as well as some lifestyle factors. Self-rated general health was assessed by the question, “How is your health right now?” Four response alternatives were dichotomized into: “good/very good” and “poor/not so good”.

Somatic health was registered by the self-reported presence of ^(1)^other musculoskeletal conditions (rheumatological disease, arthrosis, fracture/compressed dorsal vertebrae, osteoporosis and psoriasis) and ^(2)^medical conditions (cardiovascular disease, asthma, diabetes, epilepsy, kidney disease, cancer, chronic obstructive pulmonary disease and sarcoidosis). Both somatic health variables were dichotomized into no musculoskeletal or medical conditions or one or more of either conditions.

Mental distress was assessed by the Hospital Anxiety and Depression Scale (HADS), which is a validated 14-item scale that consists of two 7-item scales covering anxiety (HADS-A) and depression (HADS-D) [17]. Each item was scored on a four-point scale ranging from 0 to 3, and was added up resulting in a score between 0 and 21 for each subscale. A score of 8 or above on either subscale (recommended cut-off value) was defined as having symptoms of either anxiety or depression.

Insomnia was assessed by three questions regarding ^(1)^difficulty falling asleep at night, ^(2)^waking up too early and not getting back to sleep and ^(3)^waking up repeatedly during the night – each with the three options “never/seldom”, “sometimes” and “several times a week”. Those answering “several times a week” to one or more of the questions *in addition to* reporting sleepiness during the day were classified as having insomnia [18]. Loneliness was assessed by the question “In the last two weeks, have you felt lonely?” Four response alternatives were dichotomized into “No” or “A little/a good amount/very much”. The variable “smoking” was dichotomized and a person was defined as a smoker if reporting daily smoking of cigarettes/cigars or cigarillos/pipe. Body height and weight was measured at baseline and body mass index (BMI) was categorised into “normal weight” (BMI <25), “overweight” (BMI 25–30) or “obese” (BMI > 30). Physical activity was assessed by a physical activity index score (PAI) which is based on the responses to three questions: ^(1)^"How frequently do you exercise?" ^(2)^"If you exercise as frequently as once or more times a week, how hard do you push yourself?" and ^(3)^: "How long does each session last?" An index score from 0 to 15 was calculated. The index is thoroughly described and has been found reliable in a former study [19].

#### Sociodemographic factors

Demographic characteristics were age, sex and marital status (married/not married). Some work related factors were registered; whether or not the subject was in full-time work and whether the work involved heavy lifting. Socioeconomic status was given through the variable educational level, based on the International Standard Classification of Occupations - ISCO-88 [20]. 10 major classification groups were recoded into two levels of education: secondary and less (up to 12 years), and tertiary (13 years and above).

### Variables collected throughout the year

Current pain intensity, strongest pain intensity last month, pain- related work limitations and physical activity index score (PAI) were recorded at Q1 – Q5.

### Statistics

Descriptive statistics were used to present the characteristics of persons consulting “Conventional health care”, “Alternative health care only” or “No health care” in the 12 months following the new pain episode. Due to relatively low numbers of persons consulting alternative health care only, merely descriptive data are presented for this group. In the further analyses they were included in the “no conventional health care” group.

Logistic regression analyses were performed to study associations between explanatory factors and health care contact, using “conventional health care contacts” in contrast to “no conventional health care” as the main outcome. To account for the design of repeated measurements (Q1-Q5; Fig 1), we used logistic GEE (generalized estimating equations) models. Thus, each individual contributed with several observations (corresponding to the number of occasions participated). By specifications of the time dimension (occasions Q1 – Q5) and group level (person) the GEE analysis incorporates the longitudinal data structure and adjusts for the fact that observations on the same individual are dependent (clustered).

The analyses exploring the associations between sociodemographic factors and health care contact were adjusted for age, sex and time of follow-up (Q1-Q5). The analyses assessing the relationship between baseline somatic and mental health and health care contact were in addition adjusted for marital status, work related factors and socioeconomic status in one model, and additionally for baseline pain (current pain and strongest pain) in a separate model. The estimates from the regression analyses were used to calculate the difference in the proportion seeking health-care between the exposure groups versus the group defined as the reference measured in percent points.

We used three analytic approaches in the longitudinal analyses. First; we analysed the association of pain-related factors (current pain, strongest pain, pain-related work limitations and physical activity) with health care contact at the same occasion, adjusting for age, sex and time. Second; we assessed the associations between the abovementioned exposure factors at occasions Q1-Q4 and health care contact at the *following* occasion (Q2-Q5, lagged), adjusted for age, sex and time. This was done in order to find whether the presence of exposure factors may lead to a delayed response in terms of health care seeking. Third; we performed analyses assessing the relationship between each pain dimension and health care contact by conditional logistic regression, comparing each person at each occasion to him- or herself at the other occasions. In this analysis, only the individuals who differed in their health care contact status across the follow-up contributed with information. The resulting estimates reflect the within-person effects and are automatically adjusted for all the confounding factors that are stable within the same person within the study time frame. For the latter analyses, we presented the results as odds ratios.

Since it is reasonable to believe that the associations between baseline variables and health care contact could vary over the 1-year of follow-up (i.e. weaker associations over longer time) all analyses were performed with the inclusion of an interaction term between exposure and time.

The subjects that reported new episodes of neck and shoulder pain were analyzed separately for associations between baseline socioeconomic factors, somatic and mental health related factors and health care contact. This could not be done for the subjects with new episodes of low back pain due to lack of power. All the analyses were performed using STATA 13.1.

Power calculations were not performed for this study; all available subjects from two of the municipalities participating in the health survey (HUNT III) were included and screened. The study was approved by the Regional Committee for Medical Health Research Ethics of Central Norway (REC Central), Det medisinske fakultet, Medisinsk teknisk forskningssenter, 7489 Trondheim, Norway.

## Results

From the total of 219 individuals in the cohort, 93 persons (43 %) sought conventional health care at some point throughout the year (doctor, physiotherapist, psychologist or chiropractor). Of them, 25 persons also sought alternative care in addition to conventional, but they were included in the conventional health care group for further analyses. 18 persons (8 %) chose alternative health care only at least once throughout the year following the new incident of spinal pain. 108 persons (49 %) did not seek any kind of health care. The number of participants at Q2-Q5 was 196 (90 %), 183 (84 %), 181 (83 %) and 175 (80 %), yielding 951 observations in total and 4,3 observations per individual on average.

Tables 1 and 2 show baseline characteristics of the study population for the three health care seeking behaviour groups;” Conventional health care, “Alternative health care only” and “No health care”.

**Table 1:** Distribution of sociodemographic factors at baseline in three subgroups of persons with different health care seeking behaviors

	Conventional health care^a^	Alternative health care only	No health care
	*N* = 93	*N* = 18	*N* = 108
Age (mean, SD)	46 (11.9)	46 (11.5)	46 (11.4)
Gender (male %)	34	39	45
Married^b^(%)	58	44	63
Fulltime work (%)	60	72	62
Heavy lifting at work (%)	13	17	17
Educational level ≥ tertiary^b^(%)	30	50	50

^a^group includes those who consulted both conventional and alternative health care

^b^Missing values: married (4) and educational level (1). Percentages calculated among persons with non-missing values

**Table 2:** Pain- and health related factors at baseline in three groups of persons with different health care seeking behaviors. All numbers are given in percentage of the group population without missing information on each factor^e^

	Conventional health care^a^	Alternative health care only	No health care
	*n* = 93	*n* = 18	*n* = 108
Baseline pain intensity;”current pain”			
NRS 0-2	18	33	36
NRS = 3	26	44	23
NRS ≥ 4	56	23	41
Spinal pain location(s)			
Predominantly neck pain	48	61	56
Predominantly low back pain	33	28	38
Neck pain and low back pain^b^	18	11	6
Additional painsites			
No additional sites	56	72	65
One additional site	18	11	25
Two or more additional sites	26	17	10
Frequency of pain episodes			
Never before	14	11	18
Once a year or less	15	17	28
≥ two yearly episodes	71	67	48
Self-rated general health poor	27	6	6
Medical condition(s)^c^	27	33	24
Musculoskeletal condition(s)^c^	18	17	11
Depressive symptoms (HADs ≥ 8)	15	0	4
Anxiety symptoms (HADs ≥ 8)	24	0	9
Insomnia	10	0	5
Loneliness^d^	24	0	11
Daily smoking	28	17	13
Body Mass Index < 25 (normal)	28	22	41
BMI ≥ 25 and < 30 (overweight)	44	61	45
BMI ≥ 30 (obese)	27	17	13

^a^group includes those who consulted both conventional and alternative health care

^b^egually intense pain in both areas

^c^one or more reported diagnoses

^d^reported loneliness: a little/a good amount/very much

^e^missing values (n): frequency (9), self-rated health (1), depressive/anxiety symptoms (38), insomnia (38), loneliness (3), BMI (2)

**Fig. 2:**
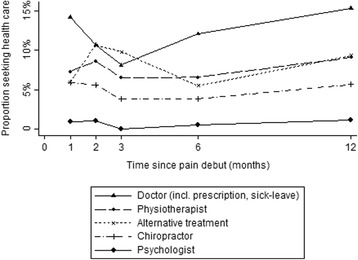
Proportion of participating subjects who at each occasion reported contact by different groups of health care professionals in the previous month

Figure 2 shows the proportion of the total population who at each occasion (Q1-Q5) reported having sought conventional or alternative health care during the previous month. Physicians’ consultations were most common at all occasions, followed by physiotherapy and chiropractor. Few individuals reported consulting a psychologist for their spinal pain. There was a decreasing trend in doctor visits the first 3 months followed by an increase to the same level as baseline at 12 months. Alternative health care showed a different trend, with an increase the first 2 months followed by a slight decrease and then stabilisation. The proportion seeking health care was stable throughout the year for the other three professions.

**Fig. 3:**
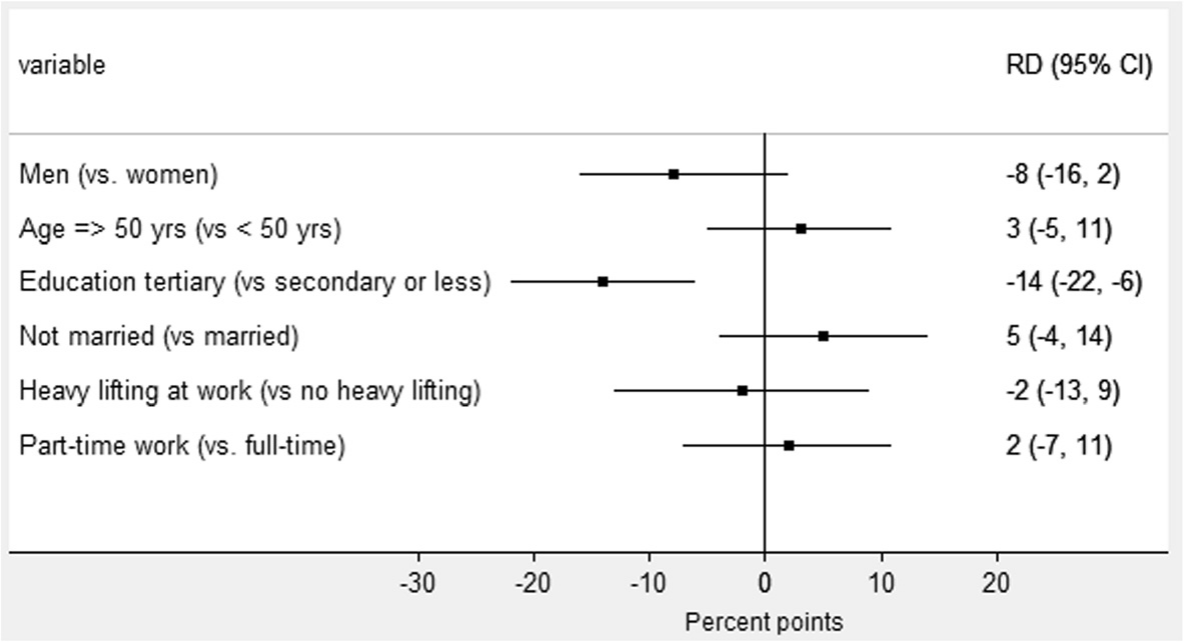
Associations between baseline factors and health care contact (conventional) for demographic and work-related factors (*n* = 921). The figure shows the predicted differences in the proportion of subjects seeking health-care (measured in percent points) between each of the different exposure groups. A difference of zero (the vertical line) indicates no association between the variable and health care seeking

**Fig. 4:**
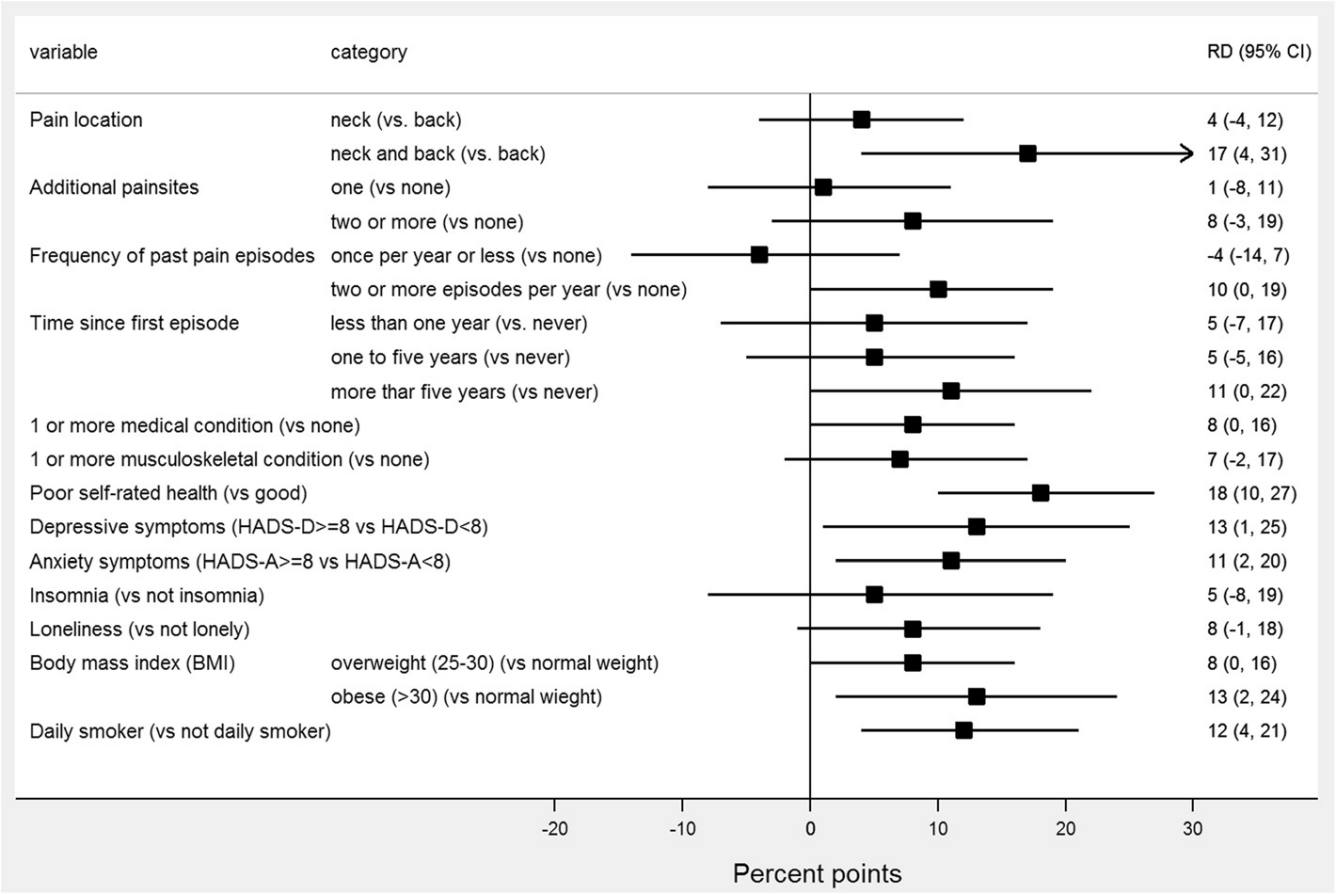
Associations between baseline factors and health care contact (conventional) for pain related and other health related factors (n varies between 795 and 921). The figure shows the predicted differences in the proportion of subjects seeking health-care (measured in percent points) between each of the different exposure groups. A difference of zero (the vertical line) indicates no association between the variable and health care seeking.

The estimated differences in health-care seeking between exposure groups are presented in Fig. 3 for sociodemographic and work-related factors, and in Fig. 4 for pain-related and other somatic and mental health factors. Participants with missing values on adjustment variables were excluded from the regression analyses (*n* = 6). For each of the health variables analyses were performed on participants with complete data on that variable, thus N varied between models. For the sociodemographic and work-related factors (Fig. 3) we found no substantial differences except for education. For tertiary education, the proportion seeking health care was 14% points (95 % CI: −22, −6) lower than for those with lower education. Figure 4 illustrates that almost all the pain related and other somatic and mental health factors were associated with health-care seeking behaviour. For instance, the proportion seeking health care was 18% points (95 % CI: 10, 27) higher among those who reported poor health at baseline compared to those who reported good health. Also, there was no indication of effect modification by time (*p*-values for interaction terms between exposure variables and measurement occasion was between 0.14 and 0.89), except for depressive symptoms. Here, the increase in the proportion seeking health care for the depressed vs the non-depressed showed a decreasing trend over time, from 33% points (95 % CI: 10, 57) at baseline to 0 at Q5, *p*-value for interaction 0.02). Estimates were generally very similar for the separate analysis of the subjects with neck and shoulder pain but statistical significance was only reached for education, daily smoking and overweight, probably due to lack of power.

Current pain intensity, strongest pain last month and pain-related work limitations were all associated with a recent health care contact, compared with those with lower levels (Table 3). A one-unit increase in current pain or strongest pain on the NRS scale (0–10) was associated with an overall 23–24 % increased odds of reporting concurrent health care contacts. When we performed the lagged analyses with the exposures at one occasion and health-care contacts at the following occasion (e.g., pain at Q2 and health care contacts at Q3), the overall associations between pain and work limitations and health-care contacts were still present, but weaker for strongest pain. In the analyses where each person at each occasion was compared with him- or herself at other occasions (Table 3, last column); a one-unit increase in current pain or strongest pain compared with that person’s pain score at other occasions was associated with a 33–40 % increase in the odds of recent health care contacts. An almost 51 % increase in odds of a recent health care contact was found for each unit increase on the pain-related work limitation index. Physical activity level was not associated with health care contact in any of the analyses.

**Table 3:** Effects of pain intensity, pain related work limitations and self-reported physical activity on health care contact throughout a year of follow-up after a new incident of neck or low back pain

	Adjusted^a^	Lagged	Within person effect^b^
	Odds ratio (95 % CI)	Odds ratio (95 % CI)	Odds ratio (95 % CI)
Current pain intensity +1 on NRS scale (range 0–10)	1.23 (1.14–1.32)	1.22 (1.11–1.33)	1.33 (1.14–1.56)
Strongest pain last month +1 on NRS scale (range 0–10)	1.24 (1.15–1.32)	1.13 (1.04–1.22)	1.40 (1.22–1.61)
Work limitations last month +1 on work limitation scale (range 0–6)	1.40 (1.25–1.57)	1.28 (1.11–1.47)	1.51 (1.20–2.84)
Physical activity index +1 on PAI score (range 0–15)	0.98 (0.91–1.05)	0.99 (0.91–1.08)	0.99 (0.86–1.15)
	938 observations 219 persons	693 observations 201 persons	

^a^Odds ratio associated with a one unit increase in each scale score; adjusted for time, sex and age group

^b^Odds ratio associated with a one unit increase in each scale score; adjusted for each person’s mean across all observations

## Discussion

In this study of health care utilisation in a general population cohort of 219 persons with a new episode of neck pain or low back pain, 43 % chose to consult conventional health care at least once throughout the following year, and 49 % did not report any health care contact in the year following the pain episode. A strong association for seeking care was found for pain intensity and pain related work limitations during the entire year of follow-up. Conventional health care contact was also found to be associated with pain of equal intensity in both the neck and the low back (as opposed to pain in just one site), poor self-rated health, symptoms of anxiety or depression, lower education, daily smoking and a body mass index above 30 (obesity). Age, sex, full-time work, heavy lifting at work, insomnia and physical activity were not associated with health care contact.

This study provides knowledge from the general population rather than from insurance or clinical populations and thus allows for a comparison between the care-seekers and the ones that did not seek care. The most important strength is the prospective design that allowed us to gain insight in health care contact behavior from the start of a new pain episode. Another strength is the meticulous recruitment of study cohort of persons reporting a new episode of spinal pain from a large general population survey. Some limitations should be noted. The main outcome measure of health care contact obtained at each occasion, i.e. whether the person had received treatment for their spinal pain the last month, completely covered the first 3 months of follow-up. However, the two last questionnaires, given at 6 and 12 months, only covered the preceding month. This may have underestimated the number of health care consultations between 3 and 12 months. However, we assume that seeking health care attributable to an incident pain episode most likely occurs within the first 3 months, for which we had complete data. Another limitation is an apparent underestimation of GP contact, possibly due to the wording of the question – “have you received treatment?” Persons who reported the use of prescription medication or sick-leave must have seen a GP or other medical doctor in Norway, but did in many cases not report GP contact. They may not have considered prescribed medication or sick leave certification as receiving “treatment” by a medical doctor. To compensate for this, prescription medication and sick leave were registered as physician contact in this study, which may have caused some overestimation of such contact as some subjects may have kept medications from previous GP visits. A third limitation is the low number of subjects seeking alternative health care. This prevented further analyses on this group. The level of missing information was low to moderate both for baseline variables and at Q2-Q5, and we believe that this did not bias our main results. This was supported by similar results in sensitivity analyses using multilevel logistic regression, which handle incomplete data (under the assumption of missing at random).

The findings of increased health care contact associated with increasing levels of pain is in agreement with the literature from cross-sectional studies on populations with acute or chronic pain [10–12, 21, 22]. Importantly, pain interfering with work was a correspondingly strong predictor for seeking care in this study. The frequency of previous pain episodes was high, only 16 % of this population reported no previous episodes of similar kinds and 61 % reported to have at least two episodes of spinal pain on average per year [6]. In other words, for most of the subjects the new pain episode was part of an episodic pain pattern, a pattern that has previously been described as common for both low back and neck pain [1, 2]. These indications of a recurrent pattern of both pain and health-care contact address a need to reflect on how we plan the interventions for this patient group. Current treatment guidelines for acute spinal pain emphasize reassurance and advice to stay active [23], while a more comprehensive and multidisciplinary approach is commonly recommended for chronic spinal pain [24]. To reduce the likelihood of long term history of recurrent and episodic spinal pain and frequent health care contacts, also taking the modest treatment effects for these kinds of disorders into account [4, 5] it is reasonable to consider a shift of focus from treatment emphasizing symptom relief to more attention on prevention of future pain episodes.

In accordance with previous studies [25, 26] we found associations between some but not all of the somatic and mental health factors and health care contact. The strongest association was found with the generic question on self-reported general health, an association found to be strong also in previous studies [12, 27]. A further exploration to substantiate what this generic variable contains seems worthwhile in future studies. In this study, the presence of one or more other diagnosed medical or musculoskeletal conditions was only marginally associated with health care contact due to spinal pain. The significant effects of daily smoking and obesity may still point to an unhealthier lifestyle among the health care seekers relative to the non-seekers.

The findings of poorer somatic health and higher levels of mental distress among health care seekers in this study indicate that care seekers have a variety of symptoms complementary to the actual neck or low back pain. Subjects in clinical studies are mainly selected from the care-seekers and may therefore be selected populations with more complex clinical pictures and in stronger need of care. However, this study shows that comorbidity is commonly present already at the start of or close to the initiation of a new pain episode, which probably reflect that the majority (84 %) had experienced similar pain previously. Failure to address the complexity of symptoms at the initial encounter with health care professionals may contribute to the recurrence of symptoms. A recent systematic review on interventions targeting psychosocial factors related to acute LBP found that research has focused mainly on pain beliefs and coping skills, and with disappointing results. The authors concluded that extended models integrating several psychosocial factors and multicomponent interventions are probably required to meet the challenge of LBP [28]. Patients with mental distress and other comorbid symptoms should thus be identified early as they might benefit from a different and more multifactorial treatment strategy from the very start (e.g. biopsychosocial approach) than those with less complex symptoms. Hill et al. [14] introduced a simple prognostic screening tool for LBP patients in order to allow for a stratified approach with better targeted treatment. Their approach was found to both improve patient outcomes and to be more cost-effective than current best practice in primary care [29]. A similar tool would be welcome also for neck pain.

## Conclusions

The main finding in this study was that people from the general population who seek health-care for a new neck or low back pain episode report more symptoms of mental distress and poor self-reported general health compared to those who do not seek health-care. They also report more intense pain with stronger work-limitations, and these factors strongly associated with concurrent health care contact throughout the year. The findings suggest that identification of complementary symptoms is relevant in the examination of spinal pain patients, even for those with recent onset of symptoms.
